# Pandemic influenza vaccine: characterization of A/California/07/2009 (H1N1) recombinant hemagglutinin protein and insights into H1N1 antigen stability

**DOI:** 10.1186/1472-6750-12-77

**Published:** 2012-10-30

**Authors:** Elena Feshchenko, David G Rhodes, Rachael Felberbaum, Clifton McPherson, Joseph A Rininger, Penny Post, Manon MJ Cox

**Affiliations:** 1 Protein Sciences Corporation, 1000 Research Parkway, Meriden CT 06450, USA

**Keywords:** Recombinant hemagglutinin, Influenza pandemic vaccine, H1N1, Baculovirus expression vector system (BEVS), Flublok, A(H1N1)pdm09

## Abstract

**Background:**

The recent H1N1 influenza pandemic illustrated the shortcomings of the vaccine manufacturing process. The A/California/07/2009 H1N1 pandemic influenza vaccine or A(H1N1)pdm09 was available late and in short supply as a result of delays in production caused by low yields and poor antigen stability. Recombinant technology offers the opportunity to shorten manufacturing time. A trivalent recombinant hemagglutinin (rHA) vaccine candidate for seasonal influenza produced using the baculovirus expression vector system (BEVS) was shown to be as effective and safe as egg-derived trivalent inactivated vaccine (TIV) in human clinical studies. In this study, we describe the characterization of the A/California/07/2009 rHA protein and compare the H1N1 pandemic rHA to other seasonal rHA proteins.

**Results:**

Our data show that, like other rHA proteins, purified A/California/07/2009 rHA forms multimeric rosette-like particles of 20–40 nm that are biologically active and immunogenic in mice as assayed by hemagglutination inhibition (HAI) antibody titers. However, proteolytic digest analysis revealed that A/California/07/2009 rHA is more susceptible to proteolytic degradation than rHA proteins derived from other seasonal influenza viruses. We identified a specific proteolytic site conserved across multiple hemagglutinin (HA) proteins that is likely more accessible in A/California/07/2009 HA, possibly as a result of differences in its protein structure, and may contribute to lower antigen stability.

**Conclusion:**

We conclude that, similar to the recombinant seasonal influenza vaccine, recombinant A(H1N1)pdm09 vaccine is likely to perform comparably to licensed A(H1N1)pdm09 vaccines and could offer manufacturing advantages.

## Background

A novel influenza A virus (H1N1) of swine origin emerged in Mexico and the United States in March and early April 2009. The virus quickly spread worldwide through human-to-human transmission resulting in the World Health Organization raising the influenza pandemic alert to the highest level (phase 6) on June 11, 2009 [1-3]. The outbreak and spread of the first influenza pandemic of the 21^st^ century challenged licensed vaccine manufacturers to rapidly mobilize and generate a prophylactic vaccine. Delivery of initial doses of vaccine to the U.S. public coincided with the second peak of the pandemic, too late to provide timely protection and highlighting the need for alternative production platforms [2,4].

Two types of licensed influenza vaccines are available in the U.S.: trivalent inactivated vaccine and live attenuated influenza vaccine [5,6], both produced in embryonated chicken eggs. The process of preparing a new vaccine seed strain suitable for growth in eggs can be quite lengthy, as it involves re-assortment between the genes of a high yielding donor virus, such as A/Puerto Rico/8/34, and the hemagglutinin (HA) and neuraminidase (NA) genes of the new influenza strain [7]. The candidate seed virus strains are then further selected for high growth capability in eggs before they can be used for the production of vaccines. This manufacturing process is not only lengthy but also limited in scalability due to its dependence on the availability of embryonated chicken eggs.

The production of purified recombinant hemagglutinin (rHA) subunit vaccines via the baculovirus expression vector system (BEVS) is a leading alternative platform for influenza vaccine manufacture. The most advanced influenza vaccine candidate produced using this technology (under the trade name Flublok®) is a trivalent composition of three rHA proteins corresponding to the full length HA proteins of the seasonally circulating influenza strains [8-11]. Clinical trials of Flublok have demonstrated that the vaccine is well-tolerated, immunogenic (as assessed by the induction of hemagglutination inhibiting [HAI] antibodies), and provides protection against drifted influenza viruses [8-10]. The rHA proteins in Flublok are produced using genetically modified baculoviruses in lepidopteran insect cells. The proteins are extracted and purified from cell pellet using a combination of filtration and column chromatography methods. Bulk vaccine can be produced within seven weeks of receipt of the HA gene sequence [9], making it an attractive platform for pandemic vaccine manufacturing as demonstrated during the initial outbreaks of H5N1 [12].

The original influenza seed viruses used for the egg-based production of the A(H1N1)pdm09 vaccine grew slowly, produced relatively low quantities of HA antigen and showed poor stability [7,13]. The rHA derived from A/California/07/2009 also revealed differences compared to other rHA proteins, as the pandemic rHA protein was more sensitive to proteolytic degradation and reacted uniquely in the single radial immunodiffusion (SRID) potency assay.

The objectives of this study were (1) to study the properties of the A/California/07/2009 rHA protein and compare it to other rHA proteins derived from seasonal influenza strains and (2) to develop an understanding of the cause of the instability observed with this antigen.

## Results

### Biochemical and biophysical characterization

**Figure 1:**
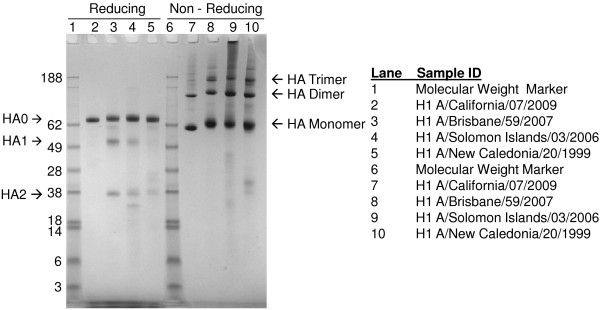
**Reducing and non-reducing SDS-PAGE of H1N1 rHA proteins. **For each sample, the respective purified rHA protein was diluted to a concentration of 100 μg/mL in reducing or non-reducing SDS-PAGE sample buffer, and 1 μg was loaded per lane. The samples were separated using 4 – 12% gradient Nu-PAGE gels and stained with Coomassie Blue. HA0 represents full-length rHA protein and HA1 and HA2 peptide fragments of HA0. Molecular weights of proteins are shown in kilodaltons. rHA proteins were produced by Protein Sciences Corporation.

The electrophoretic mobility of purified A/California/07/2009 rHA protein was compared to purified rHA derived from A/New Caledonia/20/1999, A/Solomon Islands/03/2006, and A/Brisbane/59/2007 H1N1 seasonal influenza strains using reducing and non-reducing SDS-PAGE (Figure 1). Purified rHA proteins typically migrate as monomers and disulfide-linked oligomers under non-reducing conditions. The primary full-length HA0 band migrates at approximately 62 kDa, and dimer and trimer bands are approximately 120 and 180 kDa, respectively. As shown in Figure 1, the A/California/07/2009 rHA protein displayed electrophoretic mobility comparable to that of the seasonal rHA H1 proteins. The lack of protein bands with molecular weight higher than that of HA0 under reducing conditions indicates that the oligomeric forms observed under non-reducing conditions were disulfide-linked. The A/California/07/2009 HA0 band migrated slightly faster than the HA0 comparators, possibly because A/California/07/2009 HA protein has only a single glycosylation site in the globular head (total five glycosylation sites) in contrast to the nine sites identified in the HA from A/Brisbane/59/2007 H1N1 virus [14,15]. In addition, less cleavage of A/California/07/2009 HA0 into HA1 and HA2 was observed.

**Figure 2:**
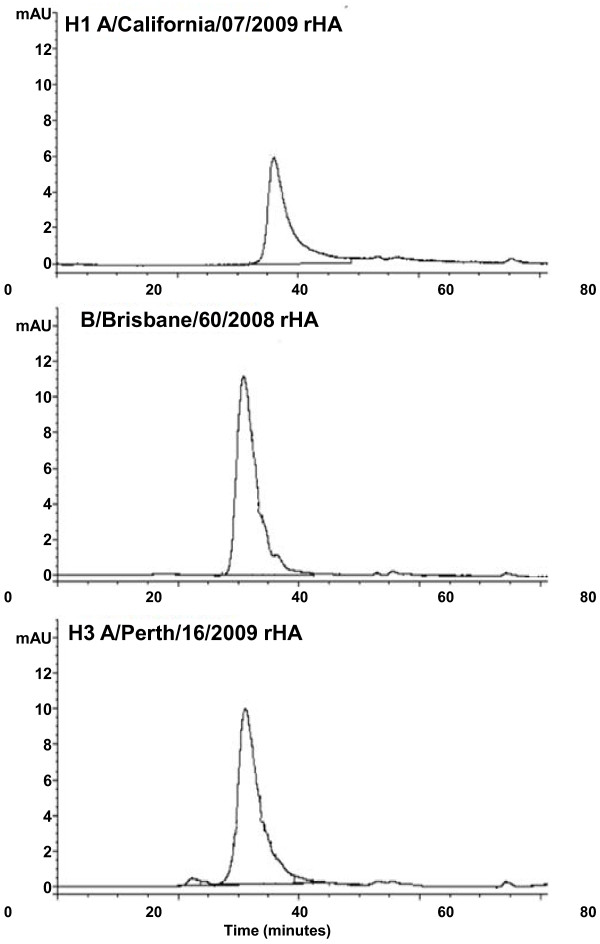
**HPLC-SEC chromatograms of rHA proteins. **Volumes corresponding to 17.5 μg of the indicated rHA proteins were injected onto a Biosuite 450 size exclusion column as described in Materials and Methods. Retention times for the respective rHA proteins were 36.0 minutes (A/California); 31.4 minutes (B/Brisbane) and 31.1 minutes (A/Perth).

A/California/07/2009 rHA protein eluted as a single peak prior to a thyroglobulin molecular weight standard (~670 kDa) similar to other rHA proteins when analyzed by HPLC-SEC (Figure 2), demonstrating that rHA proteins form high molecular weight complexes. No peaks indicative of rHA monomers, dimers, or trimers were detected. The retention time of A/California/07/2009 rHA was longer (average of 36.0 minutes) than that of B/Brisbane/60/2008 rHA (31.4 minutes) or A/Perth/16/2009 rHA (31.1 minutes). This result indicates that A/California/07/2009 rHA forms a slightly smaller complex than A/Perth/16/2009 or B/Brisbane/60/2008 rHA.

**Figure 3:**
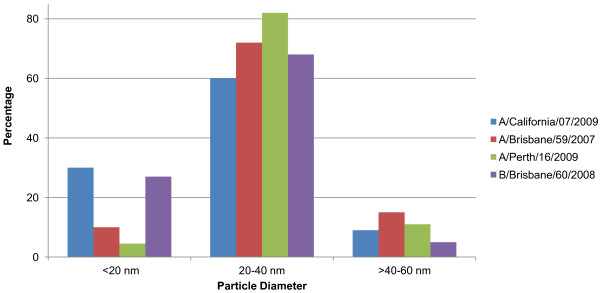
**Size determination of rHA complexes by Dynamic Light Scattering. **The results show the percentage of rHA protein in the size increments indicated. The results plotted are means of volume-average diameters in the specified size range obtained from multiple lots (A/California/07/2009 N = 5; A/Brisbane/59/2007 N = 3; A/Perth/16/2009 N = 4; B/Brisbane/60/2008 N = 4).

Dynamic Light Scattering (DLS) data showed that for all rHAs evaluated, the measured size corresponded to multimeric protein particles, with the majority falling between 20 and 40 nm in diameter (Figure 3). A greater percentage of the rHA proteins from A/California/07/2009 and B/Brisbane/60/2008 had particle diameters below 20 nm compared to A/Brisbane/59/2007 and A/Perth/16/2000 rHA. Despite these minor differences, the DLS results demonstrate that the A/California/07/2009 rHA protein forms particles with size distribution comparable to that of other seasonal rHA proteins.

**Figure 4:**
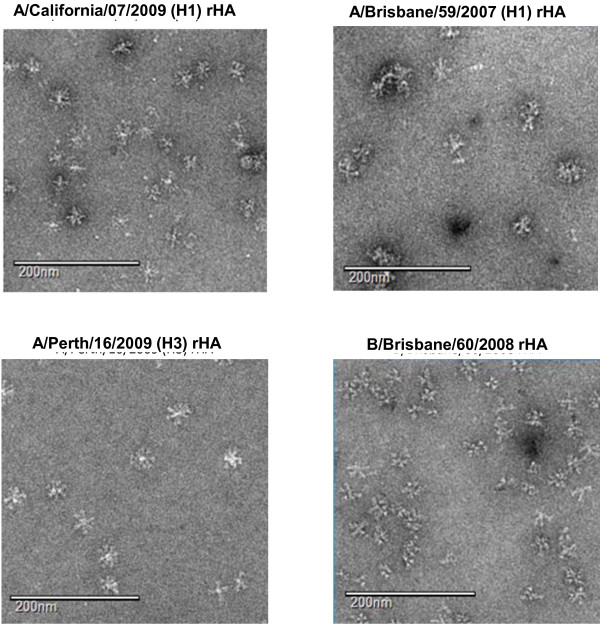
**Transmission Electron Microscopy images of purified rHA proteins. **All images were obtained at nominal 52,000× magnification. The white scale bar represents 200 nm.

Electron microscopy images of the purified A/California/07/2009 (H1), A/Brisbane/59/2007 (H1), A/Perth/16/2009 (H3) and B/Brisbane/60/2008 rHA proteins are shown in Figure 4. A/California/07/2009 rHA formed multimeric rosette-like structures consistent with the other purified rHA proteins but were less distinct compared to the H3 rHA protein, which formed clearer rosette-like structures than either the H1 or B rHA proteins. All of the rHA rosette-like structures measured approximately 30 – 40 nm in size, consistent with the measurements determined by HPLC-SEC and DLS.

**Figure 5:**
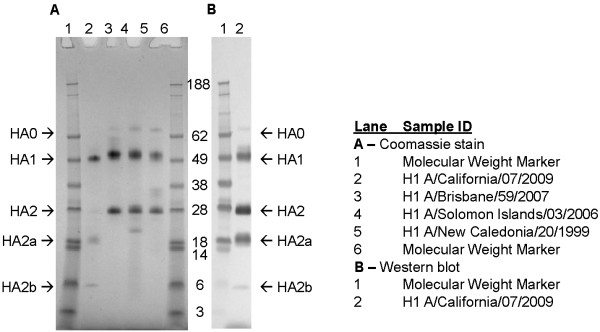
**Trypsin digestion of rHA proteins. **rHA proteins were digested with 50 μg/mL trypsin for 30 minutes at 2-8°C. Approximately 1 μg of rHA protein was loaded per lane under reducing conditions. The two additional HA2 peptides (HA2a and HA2b) are indicated with arrows. Molecular weights of proteins are shown in kilodaltons. Panel **A**: Coomassie Blue-stained SDS-PAGE gel. Panel **B**: Western blot of trypsin-digested H1 A/California/07/2009 rHA protein. rHA protein was visualized using anti-H1N1 A/California/07/2009 serum from NIBSC (lot 09/152; sheep 506/507).

Purified A/California/07/2009 rHA protein showed increased sensitivity to trypsin compared to other rHA proteins. Typically, HA0 is cleaved into HA1 and HA2 subunits when treated with trypsin, and we found that A/California/07/2009 rHA was digested into HA1 and HA2 peptides in a similar manner (Figure 5). However, we also found that A/California/07/2009 HA2 was additionally digested into two prominent peptide fragments of approximately 18 and 6 kDa (designated HA2a and HA2b, respectively), suggesting the presence of an additional trypsin proteolytic site.

In order to better characterize HA2a and HA2b and identify their cleavage site(s), the protein bands were isolated and subjected to N-terminal (Edman) sequencing. The amino acid sequencing results from the Edman analyses are shown in Table 1. The results confirm that purified A/California/07/2009 rHA is produced in its mature form (N-terminus sequence for HA0 and HA1) with a conserved trypsin cleavage site at Arginine position 324 [16]. (Note that amino acid numbering is based on full length sequence.) However, the HA2 polypeptide was found to possess an additional cleavage site at Lysine position 419 (and potential cleavage sites at Arginine-420, Lysine-426 and Lysine-427).

**Table 1:** Edman sequencing results for H1 A/California/07/2009 trypsin digested fragments

**Band ID**	**Peptide sequence**	**Cleavage position**	**Comments**
HA0	DTLxIGYHAL	A-17	Mature N-Terminus
HA1	DTLxIGY(H)A	A-17	Mature N-Terminus
HA2	GLFGAIAGFI	R-324	Conserved Cleavage Site
HA2a	RIENLNKKVD	**K-419**	Additional Cleavage Sites
	IENLNKKVDD	R-420	
	KVDDGFLDI(W)	K-426	
	VDDGFLDI(W)T	K-427	
HA2b	GLFGAIAGFI	R-324	Conserved Cleavage Site
x - unmodified Cys based on the known sequence of H1 A/California/07/2009 rHA			

N-terminal sequenced peptides were aligned with the H1 A/California/07/2009 amino acid sequence. The trypsin cleavage sites were identified based on sequence information and ExPASy Proteomic Tools (Peptide Cutter Software).

The trypsin cleavage sites are presented as conserved (for recent H1 strains) or additional cleavage sites (R-420, K-426 and K-427) and additional cleavage site K-419 is shown in bold.

**Figure 6:**
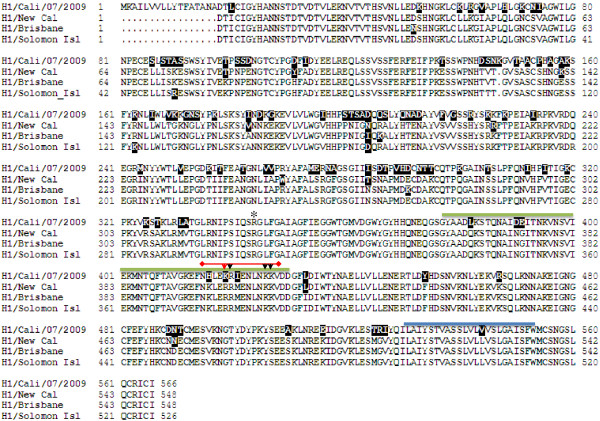
**CLUSTAL multiple sequence alignment and secondary structure prediction for H1 A/California/07/2009, H1 A/New Caledonia/20/99, H1 A/Brisbane/59/2007 and H1 A/Solomon Islands/03/2006. **A structure prediction was established using ExPASy Proteomics Tools (http://sequerome.georgetown.edu/sequerome, secondary structure prediction). The conserved cleavage site at R-324 is shown with an asterisk. The additional trypsin cleavage region is over-lined in red. The cleavage site K-419 identified from the digested fragments is indicated by a red arrow. Additional cleavage sites R-420, K-426 and K-427 are indicated by black arrows. The predicted coiled-coil region is indicated by the green over-line. The transmembrane domain is shown by the blue over-line. Amino acids that are divergent from the H1 rHA consensus sequence are highlighted in black. H1 A/California/07/2009 amino acid numbering is based on full length sequence.

Alignment of the amino acid sequences of A/California/07/2009 with A/New Caledonia/20/99, A/Brisbane/59/2007 and A/Solomon Islands/03/2006 H1 HA proteins was generated to determine whether sequences and secondary structure predictions could explain the trypsin digest results (Figure 6). The alignment results show that the three potential additional trypsin cleavage sites (i.e., Arginine-420, Lysine-426 and Lysine-427) are conserved among all four HA proteins; however, Lysine-419 is unique to A/California/07/2009. Therefore, Lysine–419 is the most likely primary protease digestion site that generates HA2a and HA2b. Interestingly, according to ExPASy proteomics tools, amino acids 377 – 427 are predicted to form a coiled coil domain (indicated by a green over-line in Figure 6). A/California/07/2009 has six amino acid changes in this region, including Lysine-419, compared to the other HA proteins. These sequence differences could affect protein conformation and make this region more accessible to trypsin digestion.

### Biological activity

A/California/07/2009 rHA protein was evaluated for functional activity by determining its hemagglutination activity using red blood cells (RBCs) and comparing it to the activities of the 2000–2010 seasonal H1 influenza strain rHAs, A/New Caledonia/20/99, A/Solomon Islands/03/2006 and A/Brisbane/59/2007. A prerequisite for hemagglutination activity is the formation of trimers and the organization of these trimers into higher order structures that can crosslink corresponding sialic acid receptors on cells. The A/California/07/2009 rHA demonstrated hemagglutination activity with guinea pig and turkey RBCs but not chicken RBCs (Table 2). This was most similar to the hemagglutination activity observed for A/Solomon Islands/03/2006 rHA with guinea pig and turkey RBCs.

**Table 2:** Hemagglutination activity of purified H1 A/California/07/2009, H1 A/Brisbane/59/2007, H1 A/New Caledonia/20/99 and H1 A/Solomon Islands/03/2006 rHA

**rHA derived from various H1N1 influenza viruses**		**Guinea pig red blood cells**	**Chicken red blood cells**	**Turkey red blood cells**
		**(HA units/μg protein)**		
A/California/07/2009	N = 8	160	<20	60
A/Brisbane/59/2007	N = 6	3200	1706	2560
A/New Caledonia/20/99	N = 2	6400	800	960
A/Solomon Islands/03/2006	N = 2	120	100	40

Hemagglutination values were generated with guinea pig, chicken, and turkey RBCs and HA activity was calculated based on protein BCA (Bicinchoninic Acid) values.

**Figure 7:**
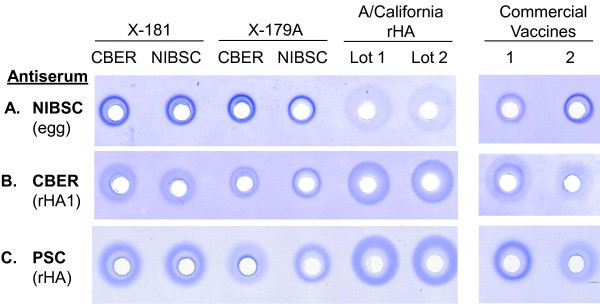
**SRID comparison of recombinant and egg-derived HA proteins and anti-serum against H1 A/California/07/2009. ** Reference antigens are identified by re-assortant (X-181 or X-179A) and source (CBER or NIBSC), and were diluted to 30 μg/mL based on the concentrations provided in the product circulars. Two licensed H1N1 A/California/07/2009 monovalent vaccines (Novartis [1] and Sanofi Pasteur [2]) were included in this analysis. The A/California/07/2009 rHA lots were diluted to a target of 30 μg/mL based on total protein measurements. Panel **A**: SRID gel using sheep antiserum obtained from NIBSC generated against egg-derived A/California/07/2009 HA; Panel **B**: SRID gel using CBER experimental sheep antiserum generated against an *E. coli*-expressed HA1 domain of A/California/07/2009 HA; Panel **C**: SRID gel using Protein Sciences Corporation (PSC) experimental rabbit antiserum generated against BEVS-insect cell derived rHA.

A/California/07/2009 rHA protein activity was also analyzed using the SRID assay. This assay measures the potency of influenza vaccines via quantification of functional HA protein [17]. Four lots of A/California/07/2009 reference antigens corresponding to re-assortants X-181 and X-179A obtained from both the Center for Biologics Evaluation and Research (CBER) and the National Institute for Biological Standards and Control (NIBSC), and two lots of the A/California/07/2009 rHA (matching Genbank accession #ACP41953) were tested against three different antisera generated against hemagglutinin from A/California/07/2009 (Figure 7). The antisera were obtained from NIBSC, CBER and Protein Sciences Corporation, and were generated using HA from egg, *E. coli* and BEVS-insect cell sources, respectively. Both the CBER and Protein Sciences antisera were experimental and produced against recombinant HA proteins (the CBER antiserum against the HA1 fragment [18] and the Protein Sciences antiserum against full length rHA).

All of the antisera produced immunoprecipitin rings with all reference antigens and the A/California/07/2009 rHA protein (Figure 7), indicating that the rHA protein was antigenically comparable. However, the NIBSC antiserum generated against egg-derived antigen produced larger, more diffuse rings for the rHA (Figure 7A) that corresponded to calculated potency values that were 2- to 5-fold greater than the amount of purified rHA protein inoculated into sample wells (data not shown). In contrast to the NIBSC antiserum, more well defined rings for rHA were achieved using antiserum generated against recombinant antigens (Figure 7B and 7C). Interestingly, both the reference antigens and two separate licensed A(H1N1)pdm09 monovalent vaccines (from Novartis and Sanofi Pasteur) reacted differently with the three antisera, suggesting a unique interaction of each antiserum with each hemagglutinin produced from the A/California/07/2009 pandemic H1N1 virus. These differences had a significant impact on the calculated potency of the commercial vaccines (Table 3) and demonstrate the need to have well-matched reagents for the pandemic H1 vaccine antigens in manufactured products.

**Table 3:** Potency by SRID for licensed monovalent H1N1 vaccines calculated using different antisera and reference antigens

**Vaccine**	**SRID reference antigen**	**Reference antigen source**	**Potency according to SRID (μg/mL)**		
			**SRID antiserum source**		
			**CBER**	**NIBSC**	**PSC**
Commercial Vaccine 1 (Novartis)	**X-181**	CBER	30.5	17.9	17.3
		NIBSC	36.6	24.4	22.2
	**X-179A**	CBER	71.4	41.0	28.4
		NIBSC	88.1	54.2	36.8
Commercial Vaccine 2 (Sanofi Pasteur)	**X-181**	CBER	15.5	14.6	17.4
		NIBSC	19.1	20.3	22.6
	**X-179A**	CBER	47.1	33.6	29.3
		NIBSC	59.1	41.8	38.8

SRID potency values are listed in μg/mL. Results were calculated using X-181 or X-179A reference antigens obtained from CBER or NIBSC, using SRID gels prepared with sheep antiserum obtained from NIBSC against egg-derived A/California/07/2009 HA, sheep antiserum obtained from CBER against *E. coli*-expressed recombinant HA1 fragment, or rabbit antiserum produced by Protein Sciences Corporation (PSC) against purified A/California/07/2009 rHA.

Finally, trivalent formulations of purified rHA vaccine corresponding to the 2008–2009 and 2010–2011 seasonal influenza strains, the latter of which contained A/California/07/2009 rHA, were prepared to compare the immune responses of the different vaccine components. A commercial egg-based 2009–2010 vaccine (FluLaval, GSK, Lot # AFLLA599BA, multi-dose formulation) was included as a control. CD-1 mice were administered two doses of the respective formulations at 21 day intervals, and hemagglutination inhibition (HAI) antibody titers were determined three weeks after each dose. The immunogenicity results are provided in Table 4.

**Table 4:** Immunogenicity of trivalent vaccine formulations

**Vaccine test article**	**Formulation ID**	**Dose (by SRID)**	**H3/Perth GMT**		H1N1 GMT*		**B/Brisbane GMT**	
			**Day 21**	**Day 42**	**Day 21**	**Day 42**	**Day 21**	**Day 42**
A/Brisbane/10/2007 (H3N2)	2008-2009 rHA Formulation	3.0 μg/HA	19.1	63.5	183.8	670.3	38.2	442.2
B/Brisbane/60/2008								
A/Brisbane/59/2007 (H1N1)		0.3 μg/HA	7.2	9.5	66.5	403.2	13.2	96.2
A/Perth/16/2009 (H3N2)	2010-2011 rHA Formulation	3.0 μg/HA	237.8	2560.0	226.3	1403.9^†^	29.7	735.2
B/Brisbane/60/2008								
A/California/07/2009 (H1N1)		0.3 μg/HA	121.8	1612.7	115.8	670.3	8.7	87.7
A/Victoria/210/2009 (H3N2)	2010-2011 Commercial Split Virion Vaccine (FluLaval® Lot # AFLLA599BA)	3.0 μg/HA	477.9	3079.7	211.1	557.1	45.9	403.2
B/Brisbane/60/2008								
A/California/07/2009 (H1N1)		0.3 μg/HA	152.7	1280.0	83.8	242.5	14.5	57.9

HAI titers were measured on serum samples collected 21 days after immunizations (Day 21 = one immunization; Day 42 = two immunizations) for the respective vaccine components and the geometric mean titer (GMT) calculated.

*HAI titers generated using matching H1N1 virus strains. ^† ^Statistically different (P < 0.01) from A/California commercial vaccine high dose group.

There was a clear dose dependence of the HAI response to all vaccine components across the test formulations, and the magnitude of the response increased from Day 21 to Day 42 after the second immunization. The immune response generated against A/California/07/2009 (H1) rHA antigen was equal to or slightly greater than that of A/Brisbane/59/2007 (H1) rHA antigen and the commercial egg-based A/California/07/2009 (H1) control by Day 42. The immune responses were also consistent for the H3 and B vaccine antigen components. These results demonstrate that rHA antigens, including A/California/07/2009 rHA, produce a robust immune response.

## Discussion

The A(H1N1)pdm09 influenza vaccine was available late and in short supply as a result of delays in production caused by low yields, poor antigen stability and absence of virus stockpile. Recombinant hemagglutinin-based vaccines are inherently less susceptible to production challenges and are a leading alternative for influenza vaccine manufacture. The most advanced recombinant influenza vaccine candidate is a trivalent formulation of seasonal rHA proteins that can be produced significantly faster than traditional egg-based influenza vaccines and has been shown to be as effective and safe as egg-derived trivalent inactivated vaccine (TIV) in human clinical trials. Pandemic rHA vaccines hold similar promise.

This in-depth characterization of A/California/07/2009 rHA showed that it is biochemically, biophysically, and antigenically comparable to seasonal rHA antigens. A/California/07/2009 rHA had an electrophoretic mobility similar to that of seasonal rHA proteins and formed higher order, multimeric rosette-like particles of approximately 20–40 nm. This is in contrast to the findings of Khurana *et al.* who expressed full length A/California/07/2009 HA in a bacterial expression system and found that the purified protein migrated predominantly as a monomer [18]. The reason for this difference is unknown but could relate to the different expression platforms. A/California/07/2009 rHA demonstrated significant biological activity and elicited a strong immune response in mice consistent with that generated by commercial egg-derived A/California/07/2009 vaccine both in this study and previously [19,20]. Together, these data support the suitability of A/California/07/2009 rHA as a pandemic influenza vaccine alternative. An initial clinical study has confirmed the safety and immunogenicity of this rHA [21].

The antigenic stability of the A(H1N1)pdm09 vaccine was found to be initially poor [13]. Trypsin digestion of purified A/California/07/2009 rHA uncovered a unique susceptibility of the protein to proteolytic cleavage not found in the seasonal rHA comparators. N-terminal (Edman) sequencing revealed that this cleavage occurs in a subdomain of the HA2 region of the protein that for most HA proteins are predicted to be structured as a coiled-coil. We postulate that the six amino acid changes in this domain in A/California/07/2009 HA may disrupt this structure, leading to decreased antigenic stability. Further studies are needed to determine whether the virus re-assortants ultimately used for egg-based A(H1N1)pdm09 vaccine manufacture possessed modifications that impacted protein structure in this region, improving antigen stability. Preliminary assessment suggests that a purified recombinant rHA derived from re-assortant virus NIBRG-121xp [7] in fact remained unstable although its interaction with sialic acid receptors was improved (data not shown).

Finally, the antigenic potency of A/California/07/2009 rHA, as determined by the SRID assay, showed dramatic heterogeneity (≥ 2-fold) depending on the assay reagents used. A similar effect was observed for licensed egg-derived monovalent vaccines. This variation indicates that preparation of reagents for potency testing (antisera and reference antigens) with novel pandemic influenza viruses requires further assessment to accommodate recombinant manufacturing strategies available for rapid pandemic response. Moreover, the development of alternative potency assays that are less dependent upon specific antigen-antibody interactions that could be affected by the manufacturing platform is warranted.

## Conclusions

These results show that the production of purified recombinant hemagglutinin (rHA) subunit vaccines via the baculovirus expression system is a leading alternative platform for influenza vaccine manufacture. The biochemical, biophysical and immunological characterization of a purified recombinant A/California/07/2009 (H1N1) hemagglutinin has been compared to different seasonal rHA proteins and egg-produced A/California reagents. The data show that purified A/California/07/2009 rHA molecules exist in high molecular weight complexes and form rosette-like particles of 20 – 40 nm in size. Biochemically, the protein exhibits hemagglutination activity and a greater sensitivity to tryptic digestion with additional cleavage in the HA2 subunit. The unique structure of this particular HA antigen may account for poor stability. The A/California/07/2009 rHA protein was found to be antigenically similar to egg-derived virus and showed immunogenicity and development of neutralizing antibody titers in mice.

## Methods

### rHA cloning and baculovirus generation

The H1 A/California/07/2009 cDNA was generated by using influenza viral RNA as a template in a reverse transcriptase PCR reaction (RT-PCR). The cDNA was cloned into baculovirus transfer vector pPSC12, and positive clones were confirmed by DNA sequencing to be identical to the H1 A/California/07/2009 reference sequence (GenBank accession # ACP41953). *Spodoptera frugiperda* Sf9 cells were co-transfected with linearized *Autographa californica* multiple capsid nucleopolyhedrovirus (AcMNPV) genomic DNA and the pPSC12 transfer vector containing H1 A/California/07/2009 cDNA by calcium phosphate precipitation. This method generated recombinant baculoviruses harboring the gene encoding H1 A/California/07/2009 by homologous recombination. Recombinant plaques were isolated and used to generate baculovirus stocks in *expres*SF+ ® (SF+) insect cells. Further details on the cloning and expression of other rHAs using this system are described elsewhere [9,22,23].

### rHA protein production

The recombinant baculovirus stock was used to produce the H1 A/California/07/2009 rHA protein. Virus inoculum from the working virus stock was added to 450 L of SF+ insect cell culture in a 600 L bioreactor at a concentration of 2% (v/v) after the insect cells reached a density of 2.0 – 2.5 × 10^6^ cells per mL. The infected culture was incubated at 28°C for 40 – 55 hours and harvested at a viability of 70 - 80%. A cell pellet was generated by centrifugation and the recombinant protein was solubilized using a buffer containing non-ionic detergent. Cells were removed by depth filtration, and the clarified extract was applied to an ion-exchange column. Recombinant HA was eluted and subsequently bound to a hydrophobic interaction column. Following elution, the protein was applied to a Q-membrane to remove any residual DNA. Finally, Q filtrate was diafiltered and the rHA protein formulated in final buffer.

### SDS-PAGE and western blot

Proteins were separated using 4-12% NuPAGE Bis-Tris Gels (Cat# NP0323, Life Technologies Corporation, Carlsbad, CA) and 1x MES Running Buffer (50 mM MES, 50 mM Tris, 0.1% sodium dodecyl sulfate, 1 mM EDTA pH 7.3). Non-reducing SDS-PAGE gel samples were prepared using 2x non-reducing disruption buffer (120 mM Tris pH 6.8, 20% glycerol, 4% sodium dodecyl sulfate, 0.2% bromophenol blue). Reducing SDS-PAGE gel samples were prepared using 2x disruption buffer (120 mM Tris pH 6.8, 20% glycerol, 4% sodium dodecyl sulfate, 0.2% bromophenol blue, 200 mM dithiothreitol). The SDS-PAGE gels were fixed in pH 1.1 fixative (25% methanol, 10% glacial acetic acid, 10% thrichloroacetic acid) for 10 minutes, followed by staining in Coomassie Blue staining solution (0.1% Brilliant Blue R, 7.7 M reagent alcohol, 1.75 M glacial acetic acid) for 60 minutes. The gels were de-stained in 10% acetic acid. For the Western Blot, the separated proteins were transferred to nitrocellulose membrane (Cat# IB3010, Life Technologies Corporation) or to polyvinylidenefluoride (PVDF) membrane (Cat# IB4010, Life Technologies Corporation) and incubated with a 1:1,000 dilution of A/California/07/2009 (H1N1) influenza antiserum (Lot #09/152, sheep 506/507, NIBSC, UK) followed by a 1:3,000 dilution of anti-sheep IgG (whole molecule) alkaline phosphate secondary antibody (Cat# A5187, Sigma, St. Louis, MO). Recombinant H1 A/California/07/2009 proteins were visualized using chromogenic alkaline phosphatase substrates, 1x NTB (nitro-blue tetrazolium chloride, Cat# 0329, Amresco, Solon, OH) and 1x BCIP (5-bromo-4-chloro-3-indolyl-phosphate, p-toluidine salt, Cat# 0885, Amresco) in chromagen buffer (2-amino-2-methyl-1-propanol, Cat# 221, Sigma) per the manufacturers’ instructions.

### Trypsin digestion

Reaction mixtures containing an rHA concentration of 250 μg/mL were incubated in the absence or presence of 50 μg/mL trypsin (Cat# T1426, Sigma) for 30 min at 2-8°C. Digestion was stopped by heating the samples in 2x disruption buffer. Samples were analyzed by SDS-PAGE and Western blotting, as described above.

### Hemagglutination assay

The hemagglutination assay was performed in U-bottom 96 well microtiter plates. Recombinant HA antigen (starting concentration of 1 μg/mL; protein concentration determined by BCA [Bicinchoninic Acid; Cat# 23225, Thermo Scientific, Rockford, IL]) was diluted by two-fold serial dilution to a final dilution of 4,096-fold. Fresh red blood cells (RBCs) were washed with 1x PBS (pH 7.2) and then added to the wells. RBCs used include 0.5% chicken RBCs from Charles River SPAFAS, Charleston, SC (Lot #A101213), 1% guinea pig RBCs from ViroMed Laboratories, Minnetonka, MN (Lot#11916), and 0.5% turkey RBCs from ViroMed Laboratories (lot#10984). After one hour of incubation at room temperature, the plates were scored for agglutination. The HA activity is defined by the dilution at which partial agglutination was observed (i.e., 50% of the RBCs were agglutinated or the pellet appeared loose). If only fully agglutinated and/or tight pellets were observed, the endpoint was defined as the average of the dilutions with agglutinated and tight pellets. If no agglutination was observed in any well, then the activity of the test article was deemed less than 20 units/μg of protein.

### Size exclusion chromatography (HPLC-SEC)

Size exclusion chromatography (HPLC-SEC) was performed by using an Agilent HPLC System (Agilent Technologies, Santa Clara, CA) with UV or diode array detectors and a Biosuite 450, 8 μm HR SEC column (7.8 × 300 mm) (Waters; Cat#186002166). Samples were run in a mobile phase containing 1x PBS with 300 mM NaCl pH 7.2 at a flow rate of 0.25 mL/min. For each monovalent bulk batch of A/California/07/2009 rHA, 17.5 μg of protein was used. Sizes of rHA multimers were estimated based on a standard curve generated using reagents from a gel filtration HMW calibration kit (Cat# 28-4038-42, GE Healthcare Piscataway, NJ).

### Dynamic light scattering

Dynamic Light Scattering was performed on purified rHA protein using a Malvern Zetasizer Nano-S (Malvern Instruments, Worcestershire, UK) according to the manufacturer’s instructions. Zetasizer software (Version 6.20) was used for data analysis. Bulk drug substance was analyzed without dilution by adding 60–70 μL of sample to a microcuvette and reading (typically) three sets of 12–14 individual scans. The volume-average size distributions were averaged and binned to <20 nm, 20–40 nm, and >40 nm.

### Electron microscopy

Transmission Electron Microscopy was performed by Nanoimaging Services (La Jolla, CA). Samples were prepared using a continuous carbon grid method with grids of nitrocellulose supported 400-mesh copper. Three microliters of 1:100 diluted samples (~6 μg/mL protein) were applied to a cleaned grid, blotted with filter paper, and immediately stained with uranyl formate. Electron microscopy was performed using an FEI Tecnai T12 electron microscope, operating at 120KeV, equipped with an FEI Eagle 4 K × 4 K CCD camera using nominal magnifications of 110,000x (0.10 nm/pixel), 52,000x (0.21 nm/pixel), and 21,000x (0.50 nm/pixel) at electron doses of approximately 10–15 e/Å^2^. Negative stain grids were transferred into the electron microscope using a room temperature stage.

### Edman sequencing

Approximately 6 μg of trypsin treated or untreated A/California/07/2009 rHA was loaded on an SDS-PAGE gel and run under reducing conditions. Protein bands were transferred to a PVDF membrane and stained with 0.1% Coomassie Blue R-250 in 40% methanol and 1% acetic acid, followed by de-staining with 50% methanol and water. Protein bands of interest were excised from the PVDF membrane and submitted to the Protein Core Facility at Columbia University (New York, NY) for N-terminal (Edman) sequencing. The stained bands were sequenced on an Applied Biosystems 494 protein sequencer according to the manufacturer’s instructions.

### Single radial immunodiffusion (SRID) assay

SRID assays were performed as described previously [17,24]. Briefly, an antibody solution at the optimal working concentration was mixed with melted 1% agarose (Cat# 50010, SeaKem ME, Lonza, Rockland, ME ) in 1x PBS (pH 7.2) (Cat# 20012–050, Life Technologies Corporation) at 54° - 56°C. Following solidification on GelBond film (Cat# 53734, Lonza, Rockland, ME) at room temperature, 4 mm wells were punched in the gels. Initial dilutions of test samples and reference standards were prepared in 1% Zwittergent 3–14 (Cat# 693017, Calbiochem, Darmstadt, Germany), incubated for 30 min at room temperature, and further diluted with 1% Zwittergent 3–14 in PBS. Twenty microliter samples were then applied to the agarose wells. The gels were placed in a sealed moist chamber at room temperature for 18 hours. Following incubation, the gels were washed first with 1x PBS (pH 7.2) and then water, dried and stained with Coomassie Brilliant Blue R250 (Cat# BO149, Sigma). After de-staining, the gels were dried, and SRID ring diameters were measured using the GT Vision SRID Reading Program (GT Vision LLC, Hagerstown, MD). The diameters of the precipitin rings were measured in two orthogonal directions. Recombinant HA potency was calculated in μg/mL by the parallel line bioassay method using reference and test rHA antigen response curves (log antigen dilution vs. log zone diameter). Statistical parameters for determining test validity were based on correlation coefficients (r ≥ 0.95) and the equality of slopes (t < 4.604) between test and reference antigens. NIBSC antiserum lot 09/152, CBER experimental antiserum lot H1-Ab-1004 and Protein Sciences Protein G purified rabbit antiserum were used in the SRID assay. In addition, X-181 CBER antigen lot H1-Ag-1002, X-181 NIBSC antigen lot 09/294, X-179A CBER antigen lot 69, X-179A NIBSC antigen lot 09/174, and two different purified rHA lots were used. Licensed (egg-derived) monovalent A/California/07/2009 H1N1 pandemic vaccines from two manufacturers were also evaluated (Sanofi Pasteur Lot UP088AA and Novartis Lot 110739).

### Generation of rabbit polyclonal antibody

New Zealand White Rabbit immunization was performed under contract with Harlan Bioproducts for Science, Inc. (Madison, Wisconsin). An approved 112 day animal protocol was used. Recombinant H1 A/California/07/2009 was used as an antigen. Freund’s adjuvant was used to generate the rabbit antiserum. Approximately 2 mg of antigen at a minimum protein concentration of 0.5 mg/mL was used per rabbit. The rabbit antiserum from the final bleed was purified over a Protein G Sepharose column (Cat# 17-0618-05, GE Healthcare) per the manufacturer’s recommendations. The eluted antibody fraction was dialyzed against 1x PBS (pH 7.2) (Cat# 20012–050, Life Technologies Corporation). The purified rHA H1 A/California/07/2009 antibody was used in the SRID assay. All work was conducted ethically, with animal welfare as a top priority, in full compliance with national animal welfare regulations, under PHS Assurance in a fully USDA licensed facility.

[http://www.harlan.com/about_harlan_laboratories/animal_welfare.hl].

### Animal immunization and HA inhibition (HAI) assays

The Animal Core Facility at Colorado State University (CSU) conducted the immunization and determination of HAI titers. Briefly, 6 – 8 week old female CD1 mice were administered trivalent vaccine formulations containing purified recombinant H1 A/California/07/2009 rHA, H1 A/Brisbane/59/2007 rHA, or an A/California/07/2009 egg-based commercial vaccine in trivalent formulation (FluLaval®, GSK, Lot # AFLLA599BA, multi-dose formulation) by intramuscular (IM) injections. The formulated doses were based on SRID.

For HAI titer determination, individual serum samples were treated with receptor destroying enzyme (RDE, from *Vibrio cholera* Denka-Seiken, Tokyo, Japan) to remove non-specific inhibitors and tested against 4 hemagglutination units (HAU) of the respective influenza viruses grown in eggs using 0.5% chicken RBCs as previously described [25]. All serum samples were tested in duplicate at a 1:10 starting dilution. The HAI titer was defined as the reciprocal of the greatest dilution that completely inhibited the agglutination of the chicken RBCs. A titer value of 5 was assigned to represent responses below the assay detection limit. All work was performed ethically in compliance with all federal, state, and local laws, regulations, and policies, as well as Colorado State University internal policies http://web.research.colostate.edu/ACP/Regulations.aspx.

## Competing interests

All authors work for Protein Sciences Corporation, which has a financial interest in recombinant influenza vaccine.

## Authors’ contributions

EF carried out H1N1 process development, protein purification, rabbit antibody production, hemagglutination assay, trypsin digestion, Edman sequencing and helped to draft the manuscript; DR carried out Dynamic Light Scattering, participated in design of the animal study, EM and helped to draft the manuscript; RF drafted the manuscript and revised the paper; CM coordinated the HPLC-SEC, SRID assay and helped drafting the manuscript; JAR and PP designed and coordinated the study and helped to draft the manuscript; MMJC supervised the project, participated in its experimental design and data interpretation, and was responsible for writing the manuscript. All authors read and approved the final manuscript.
